# Perspectives of Mauritians living with neurological disability and their family caregivers during the COVID-19 lockdown: a thematic analysis from an African nation

**DOI:** 10.11604/pamj.2025.52.57.48098

**Published:** 2025-10-02

**Authors:** Britney Angelica Soll, Vincent Oxenham, Sudhir Kowlessur, Farris Kassam, Ori Bhoosun, Rajiv Reebye

**Affiliations:** 1 New School of Psychotherapy and Counselling, London, United Kingdom; 2 Canadian Advances in Neuro-Orthopaedics for Spasticity Consortium, Vancouver, Canada; 3 School of Psychological Sciences, Macquarie University, Sydney, Australia; 4 Department of Neurology, Royal North Shore Hospital, Sydney, Australia; 5 Non-Communicable Diseases, Health Promotion and Research Unit, Ministry of Health and Wellness, Emmanuel Anquetil Building, Port Louis, Mauritius; 6 Ministry of Health and Wellness, Emmanuel Anquetil Building, Port Louis, Mauritius; 7 Division of Physical Medicine and Rehabilitation, Department of Medicine, University of British Columbia, Vancouver, Canada

**Keywords:** Disability, stroke, spinal cord injury, traumatic brain injury, Mauritius

## Abstract

**Introduction:**

Mauritians living with neurological disability face limited medical care, accessibility issues, and cultural stigma, which prevent community reintegration. We aimed to investigate the perspectives of Mauritians with neurological disability and their family caregivers during COVID-19 lockdown.

**Methods:**

following a phenomenological epistemology, this qualitative study employed an inductive reflexive thematic analysis of semi-structured interviews with neurorehabilitation patients (N=18) and their primary caregivers (N=22). Patients with stroke (N=22), spinal cord injury (N=11), and traumatic brain injury (N=1) were included, excluding those who did not experience cognitive and/or physical disability.

**Results:**

the analysis developed six themes: (1) caregiver burnout: “I do everything”; (2) barriers to treatment: “the kind of care we get”; (3) poor social support: the disability taboo; (4) geographical barriers: a world not built for you; (5) financial burden: “sinking us into a hole”; and (6) isolation: a double-sided coin. Unexpectedly, lockdowns benefitted socially isolated patients, resulting in strengthened familial bonds, subjective gains in recovery, fewer experiences of stigma, and reduced fatigue. Rehabilitation care is limited, and patients lose motivation to pursue medical care. As a result, medical care was not highly affected by the COVID-19 lockdowns. Instead, shortages and rising prices of medication posed issues.

**Conclusion:**

the widespread lack of resources for patients on financial, rehabilitation, and social levels prevents patient return to the community and causes high rates of caregiver burnout. We recommend patient-centric strategies at the grassroots, community, and national levels to enhance accessibility, improve inclusivity, support caregivers, and reduce barriers to neurorehabilitation services.

## Introduction

Mauritius is home to a culturally diverse islander nation of 1.27 million people [1]. The island experiences high rates of traumatic brain injury (TBI), spinal cord injury (SCI), and stroke, with stroke incidence increasing by 80% between 2009-2019 [2]. While the provision of neurorehabilitation services has accelerated in recent years, the country continues to face limitations in financial and human resources, leaving rehabilitation professionals unable to meet the high and growing demand. These systemic constraints, combined with cultural barriers typical of developing countries [3], result in poor reintegration of individuals with neurological disabilities into the community [4].

Developing countries face a disproportionately higher burden of neurological illness due to poverty and lack of appropriate medical care [5]. The literature further illustrates that cultural practices, superstitious beliefs, and social stigma impede the delivery of neurological care in these contexts [6-9]. During the COVID-19 pandemic, individuals with neurological injuries experienced worsening access to medical care, diminished community support, and increased psychological distress [10-12]. However, these studies have largely focused on populations in high-income countries, which differ significantly in both healthcare infrastructure and cultural paradigms.

To our knowledge, this is the first qualitative analysis of Mauritians with neurological injuries. Although the present study was originally designed to investigate the impact of lockdowns on these individuals and their family caregivers, participants´ narratives revealed enduring difficulties that extended well before and long after the pandemic period. As such, the study offers insight into the broader and ongoing challenges of living with neurological disability in Mauritius outside the scope of the pandemic. The current analysis aimed to understand how participants make sense of their embodied, relational, and contextual experiences and how these were impacted by the COVID-19 lockdown.

## Methods

**Qualitative approach and research paradigm:** Reflexive Thematic Analysis (RTA) was used to analyse the interview transcripts, as it produces an organic interpretation that evolves throughout the writing process [13,14]. The phenomenological epistemology reflected our aim to provide comprehensive insight into the daily lives of this underrepresented group by examining phenomena that appear in their lived experience [15].

**Researcher characteristics and reflexivity:** among the authors are four PhD-level academics (RR, VO, SK, BO) from multiple disciplines (physical medicine and rehabilitation, neuropsychology, public health and epidemiology), one medical student (FK), and one counselling psychology student (BS). All except one are either Mauritian or have lived on the island for over half their lives, allowing the authors to connect with participants on a cultural and dialectal level. As we live without neurological disability, we acknowledge that our perspectives may be tainted by ableist views. However, since two of the authors work with neurologically injured patients daily, they may be well-placed to consider factors that provide a deeper understanding of the challenges faced by the participants.

**Context:** due to COVID-19 conditions, tele-interviews were primarily conducted. However, in-person interviews were conducted upon participant requests and when lockdown protocols were lifted.

**Sampling strategy:** we used an opportunity sampling strategy [16] to recruit patients with self-reported physical and/or cognitive disability resulting from SCI, TBI, or stroke. Patient lists were sourced from private practice neurologists, non-governmental organisations, and medical equipment suppliers. A cold call strategy resulted in a 60.71% success rate. Patients and family-caregivers of patients without physical or cognitive disabilities were excluded.

The study included all eligible participants who were available and willing to participate during the data collection period. Consequently, the sample size was limited by pragmatic constraints and participant willingness, rather than by a pre-specified target. Although data saturation is not a methodological requirement within a phenomenological epistemology, we consider that a point of sufficient depth and richness in the data was nonetheless achieved as the same themes began to resurface in later interviews. The final sample included 18 patients and 22 family caregivers. A total of 34 interviews were conducted with either solely the patient (16), solely the caregiver (6), or with both the patient and the caregiver (12). Where only the caregiver was interviewed, the patient demographic data were included (Table 1).

**Table 1:** demographic characteristics of study participants living with neurological disability (N=34), recruited from private practice neurologists and non-governmental organisations (NGOs) in Mauritius, from August 2021 to February 2022

	Frequency	Percentage	Mean	SD
**Age**			**52.41**	**18.94**
11-30	6	17.65%		
31-50	8	23.53%		
51-70	13	38.24%		
71-90	7	20.59%		
**Sex**				
Female	9	26.47%		
Male	25	73.53%		
Ethnicity				
African	5	14.71%		
Indian	25	73.53%		
European	2	5.88%		
Chinese	2	5.88%		
**Etiology**				
Stroke	22	64.71%		
Traumatic brain injury	1	2.94%		
Spinal cord injury	11	32.35%		
**Year acquired (years since injury)**			**6.53**	**9.73**
2020-2021	9	26.47%		
2015-2019	15	44.12%		
2010-2014	6	17.65%		
2005-2009	1	2.94%		
<2005	2	5.88%		
**Cause of injury**				
Fall	4	11.76%		
Disease	23	67.65%		
Vehicular accident	6	17.65%		
Other physical injury	1	2.94%		
**Education**				
None	2	5.88%		
Special needs	2	5.88%		
Primary school	6	17.65%		
Intermediate	16	47.06%		
Tertiary	8	23.53%		
**Marital status**				
Single	8	23.53%		
Relationship, unmarried	1	2.94%		
Married	22	64.71%		
Divorced	3	8.82%		
**Primary caregiver**				
Partner	16	47.06%		
Parent	10	29.41%		
Son/daughter	2	5.88%		
Other family member	4	11.76%		
Caregiver (not family)	1	2.94%		
No caregiver (self)	1	2.94%		
Size of family in household			3.41	1.26

**Ethical issues pertaining to human subjects:** informed consent was obtained verbally from participants before the interview was conducted. They were informed that they could end the interview at any time and that they could withdraw their data from the study for up to two weeks following the interview. Ethical approval for this study was obtained from the Mauritius National Ethics Committee. Project Protocol: MH/CT/NETH/2021 V3.

**Data collection methods:** data was collected between August 2021 and February 2022. Interviews lasted between 15 minutes and 2 hours (M=33 mins, SD=15 mins). The researcher explained the purpose of the research, and participants were encouraged to ask questions before agreeing to take part. Most participants questioned the researcher´s reason for contacting them and agreed to participate when informed that the researchers collaborated with the Mauritian Ministry of Health and Wellness. Interviews were audio-recorded once participants provided verbal consent and were reminded of withdrawal procedures. Based on participant preference and the ability of the interviewer (first author, BS), interviews were conducted in a mixture of French, English, and Mauritian Creole.

**Data collection instruments and technologies:** the semi-structured interview schedule comprised two parts. Section A assessed patient demographics and the nature of their injury (Table 1). Section B was based on the Quality of Life After Brain Injury (QOLIBRI) questionnaire [17], a cross-culturally validated tool consisting of 58 questions that produces a comprehensive overview of six functional areas, including cognition, the self, autonomy in daily life, social relationships, emotions, and physical problems. Interviews were conducted via a telephone or WhatsApp call with the interviewer and recorded using Voice Memos. NVivo software was used for coding the data.

**Data processing:** the recordings were translated, deidentified, and transcribed into British English. Where needed, transcriptions were verified by a native speaker of Mauritian Creole. Following transcription, audio recordings were deleted. All data is stored in password-protected folders in Dropbox, and deidentified transcripts are uploaded to NVivo.

**Data analysis:** an inductive RTA was used to analyse the interview transcripts, as it produces an organic interpretation that evolves throughout the writing process [13]. Coding was conducted inductively using NVivo software and revisited in iterative cycles to maintain reflexivity and consistency with the study´s phenomenological stance. A significant methodological point is that the themes encompass both shared topics (i.e., treatment issues) and shared meaning, which is not typical of RTA. Braun *et al.* [15] emphasize the importance of thoughtful practice in TA, and we believe that these choices produce a holistic picture of the daily struggles faced by participants during lockdown. This objective was also reflected in the development of six overarching themes.

**Techniques to enhance trustworthiness:** triangulation was sought through discussion with a general practitioner who has worked with neurologically injured patients and their caregivers in Mauritius for over 20 years. Her clinical insights helped to contextualise participant narratives and assess the plausibility and relevance of emerging themes. In addition, themes were iteratively discussed, triangulated, and refined through regular discussion between the authors.

## Results

**Figure 1:**
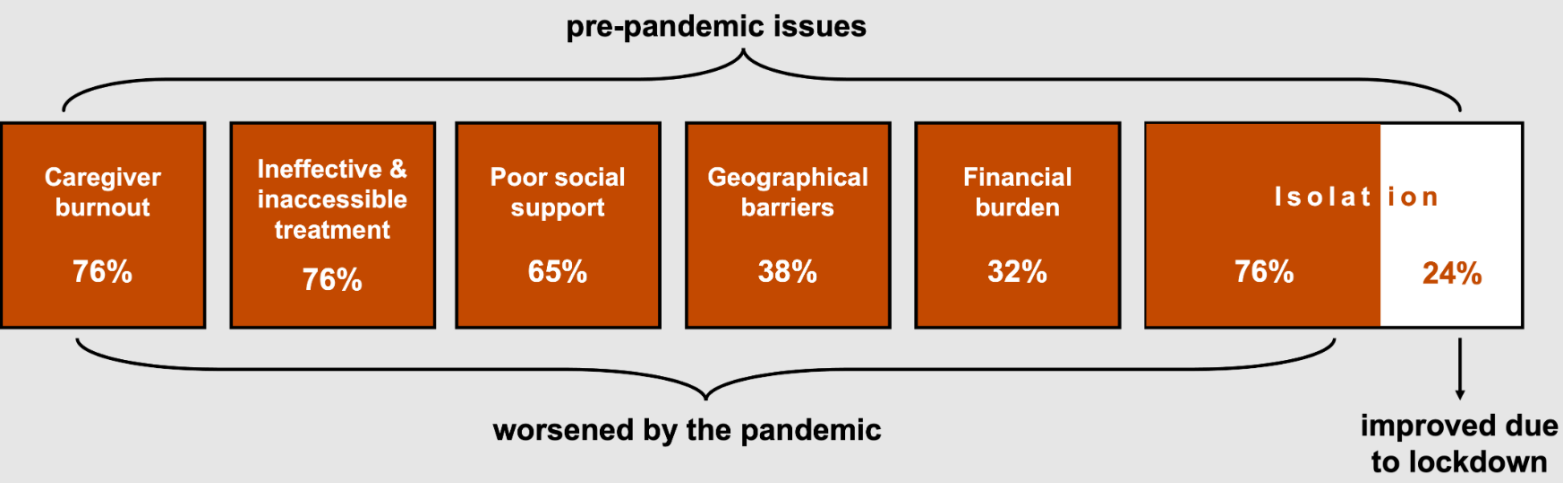
six themes produced during the thematic analysis, and whether the lived experiences of Mauritians with neurological disability and their family caregivers improved or worsened in these areas during the COVID-19 pandemic

Six themes describing participant experiences before and during COVID-19 lockdowns were developed from the data: (1) caregiver burnout: “I do everything”; (2) barriers to treatment: “the kind of care we get”; (3) poor social support: the disability taboo; (4) geographical barriers: a world not built for you; (5) financial burden: “sinking into a hole”; and (6) isolation: a double-sided coin. All six themes are linked by their existence before and during the pandemic (Figure 1). Importantly, this highlights that the pandemic put pressure on existing issues rather than creating new ones.

**Caregiver Burnout: “I do everything”:** the theme caregiver burnout describes how the burden of patient care impacts the identity and quality of life of family-caregivers. All participants lived at home, and 70% depended on at least one person to assist in their activities of daily living. As a result, caregiver burnout emerged as the most prevalent theme. Caregivers feel obligated to endorse and be “everything” to patients who can do “nothing”. The wife of a stroke patient shared, *“He needs help with everything from walking and bathing to doing activities, eating, taking care of finances. He cannot do anything alone”*. As much as the injury reshapes the patient´s life, it also marks a turning point for family members, who, as the sole source of support, feel responsible for their care. *“Well, it´s my wife! She does nothing. I have to do everything, I do all the household tasks, I do everything”*. While these roles are often taken on out of love, they are also borne of duty, not by choice.

Family members dedicate themselves to around the clock care, and their identities become engulfed by an onslaught of new roles: nurse, therapist, financial provider. One caregiver confided, *“It´s as if I do not exist”*. The wife of a paraplegic patient described her loneliness; *“Everything has changed. Sometimes I want to go out, and I want to do things. I can´t stay between four walls every day. There is no one with me to even talk to”*. Lack of social and financial support forces families into a “battle to find a solution” as they stretch their personal reserves. The 72-year-old father of a near-vegetative son shared, *“I cannot continuously help him like this […] I am very tired and with the age and all the pain”*. Despite his exhaustion, he continues on, because there is no alternative.

Caregivers who were accustomed to going about daily life outside the house reported more loneliness than patients during the COVID-19 lockdowns. Their usual outlets - the chance to “breathe” - were lost. *“I need to go out, get some air, I was suffocated”*. This sense of entrapment was exacerbated by rising living costs, medication shortages, limited medical care, and increasing social isolation - all burdens that often fell squarely on the caregiver´s shoulders.

**Barriers to treatment: “the kind of care we get”:** alongside the emotional and physical toll on caregivers, participants described major obstacles in accessing consistent and effective treatment. This theme captures the barriers impacting neurorehabilitation care and health-seeking behaviors before and during the COVID-19 lockdowns.

Learned helplessness - where repeated exposure to uncontrollable adversity leads individuals to stop trying to improve their situation - was evident. Patients and caregivers are repeatedly told to accept their condition: *"I know I will not change"*. Nearly half described treatment as ineffective: “*We didn‘t see that it amounted to anything big"*. Instead of motivating patients to pursue rehabilitation, doctors inform them that they "will not change," there is “nothing” they can do, and they "must accept their condition". Patients feel indignant, *“this is the kind of care we get”*. Feeling “neglected” by the healthcare system and “stagnant” in their progress leads to lower engagement with healthcare services over time. The husband of a stroke patient, frustrated by their inability to help, expressed, *“We need better rehabilitative services to follow her”*.

During the pandemic, fear of contracting the virus deterred treatment-seeking behaviour: *“We go out, we get problems”*. Another barrier was overstretched hospitals, which often “blocked” access to care. About 45% of participants reported not receiving medical attention during lockdown due to transport issues, restricted movement, or being turned away at the hospital. Yet 35% stated that their care was largely unaffected. Finding that hospital services were either inaccessible or ineffective (pre-pandemic), many had already adapted to relying on home-based strategies, *“the treating doctor told us we must just continue at the house”*. For many, the most critical issue during lockdown was not care interruption but shortages and rising costs of medication, *“I´m not scared of COVID-19, my pain is 10/10… when I don´t have my medications, I´m dead. That´s the problem”*. In the face of excruciating neuropathic pain and already minimal medical care, medication access took precedence over appointments because, confinement or not, the pain is there.

**Poor social support: the disability taboo:** in addition to systemic barriers, many participants spoke about the deep social stigma and isolation they faced within their communities. This theme reflects the cultural barriers that prevent reintegration, including a persistent social taboo surrounding disability. Families described varying degrees of exclusion from friends and relatives following the onset of disability, *“no one comes to see us, we are completely estranged from our friends and family. I can‘t tell you why this happened to us, but we are all done. Maybe the injury, maybe the stroke. People are scared of us”*.

Disability makes others - sometimes even close loved ones - nervous or uncomfortable. As one patient shared, *“I lost all my friends, even my girlfriend no longer wanted to be with me”*. Some laughed bitterly when asked about friendship. In addition to the unconsented loss of physical and/or cognitive function, it is unfair that they lose their loved ones as well. While some drew strength from religious belief, *“I have a big someone with me, and because of this I am scared of nothing”*, others were seen as cursed. The mother of two sons with neurological disabilities recalled, *“even my parents said it´s a curse that they are handicapped. They aggressed me. I don´t see my children as a curse”*. Rather than support, these families face rejection and anguish. The result is an implicit division between the abled and disabled, an unspoken social ruling.

Stigma breeds shame. Patients not only rely on family for physical care but also on society to accept them in social and professional roles. A customs officer worries that he will lose his job because of his limp; *“I see the future very darkly now, my life depends completely on others”*. Similarly, another stroke patient´s wife reports that he struggles to meet new people as he is judged for the permanent “grimace” on his face. During the pandemic lockdown, some patients preferred walking outside, away from the “strange looks” of others, *“it hurt me to go out because people see me as handicapped”*. Others described the “pointed” and “funny” looks from others. Leaving home subjected them to scrutiny, making them feel conspicuously out of place.

**Geographical barriers: a world not built for you:** these social exclusions were mirrored by physical and infrastructural challenges that further restricted independence and integration. The theme “geographical barriers” includes barriers to reintegration, including housing, public infrastructure, and transport. Patients must navigate a physical world that does not accommodate them; the barriers of the physical world echo the message from the social world: we are not here to accommodate you. In a place that is meant to be their home, wheelchair-users are isolated, struggling to access bathrooms and move between rooms in the house, *"He bathes in the lounge, he works in the lounge, he eats in the lounge, but the lounge is a place to live. He doesn‘t have a space for himself"*. With narrow halls and awkward bathrooms, even their own homes become treacherous, *"the bathroom is not safe for him. He broke his femur trying to get into the bath"*.

Stepping outside their homes, the local infrastructure is even less accommodating: broken sidewalks, potholes, and cars parked on the sidewalk make it difficult to navigate through town, while narrow doors, a lack of ramps, and bus stairs impede easy access to public spaces, *"say our family goes to the cinema, we need someone to push the chair and to bring him inside, because there are only stairs, there aren‘t ramps at all"*. Patients are provided with bulky, old-fashioned wheelchairs, which are difficult to maneuver. The wife of a stroke patient who struggles to access public spaces is exasperated by the effort required to perform daily tasks: *“at the dentist, he couldn‘t get through the doors, he had the treatment outside. They gave him the services outside. Because he couldn‘t get in. Even the dentist didn‘t understand how you could have a chair that does not fit inside the room”*. Lockdown benefited certain employed patients by removing the fatigue of transport, *"in terms of work from home, you are more relaxed, don‘t have the stress of transport, it‘s much more comfortable for me to work from home"*. No longer battling social and physical barriers that impede integration into the social world, they can blend into an online space where physical injuries are less apparent.

**Financial burden: “sinking us into a hole”:** compounding these challenges, participants also highlighted the financial burden brought on by neurological disability. Many families not only lose income from a contributing member but also incur costs of medication, therapy, and equipment that come with neurological injuries. Depending on their circumstance, family caregivers may retire early and rely on savings to provide 24/7 care for the patient. In other families, homemakers take on new financial responsibilities. The wife of a stroke patient describes the strain of financial burden, *"they say that money is not happiness, but that is a lie, for without money there is no happiness. Without good health, there is also no happiness. Money and health are everything"*. The COVID-19 pandemic increased financial burden across the globe. For these families, additional financial burdens included: inaccessible public transportation leading to the use of increasingly expensive private transport, as well as steepening costs of living and medication, and loss of employment and income. One patient describes the system as, *“sinking people like us into a hole [...] not to give us a hand to help us come out of this hole. Do you understand? It is very very very difficult. We do not agree with this”*. The family caregivers who bear the financial burden of care find that they are sinking into a hole with no help on the horizon. The position they are in was not predicted, consented to, nor does it seem to have an end. However, the pressure of the pandemic inspired one cognitively able paraplegic patient to take on new projects to contribute to household income, *“we needed money, we didn‘t have enough. I tried to look for projects to do after confinement to contribute [...] I became more motivated to look for work”*.

**Isolation: a double-sided coin:** participants reflected on how isolation during COVID-19 brought both psychological distress and, for some, unexpected moments of connection. This theme captures contrasting experiences. While 76% of cases reported negative effects due to increased isolation, about a quarter described improved mood - often shaped by whether their routines were disrupted or maintained. Some patients experienced intense emotional reactions: *”He was nervous, he was angry, he had rage, he felt trapped [...] we´ve gone backwards with everything, we struggle to get up and going again. With all the things that build up, it´s hard to stand up”*.

Loss of routine and social isolation left patients “demoralized” with increased symptoms of anxiety and depression. One caregiver observed, *“She had a lot less energy during confinement”*, while another noted reduced cognitive function, *“She takes her time to understand us”*. Some patients lost what little support remained, *“the children could no longer come, this affected us because we were alone”*. One stroke patient explained, *“the little control I had over things I could do was taken away from me”*, while another lamented not being able, *“to do at least the minimum one could do when not in confinement”*. The pandemic had stripped them of their remaining autonomy: *“when there is no confinement, I have just a little control [...] having to stay in the house took away the little control I had”*, and forced them into another situation to which they did not consent, *“we are angry that we can do nothing”*.

In contrast, 24% reported gains during lockdown. Increased contact with family led to improved mood, speech, and cognitive function. Many were already isolated pre-pandemic, *“I didn´t use to see many people before. I just do nothing [...] it was not worse during the confinement; everything is the same for me”*. With family at home, they received more support: *“We were more at the house [...] we could do more physio with him. We are well surrounded”*. Another shared, *“because everyone has been with her, she has become happier and more affectionate”*. These six themes collectively show that the pandemic did not create these challenges - it amplified longstanding systemic, structural, cultural, and emotional barriers.

## Discussion

This study explored the lived experiences of Mauritians with neurological disabilities and their family caregivers during the COVID-19 lockdown. Six key themes emerged: caregiver burnout: “I do everything”, barriers to treatment: “the kind of care we get”, poor social support: the disability taboo, geographical barriers: a world not built for you, financial burden: “sinking into a hole”, and isolation: a double-sided coin. Lezak [18] cite the first-world issue of needing to convince family members to participate in rehabilitation. In stark contrast, our findings indicate that families are often the only support for patients post-injury. It is estimated that in India, high caregiver burden is experienced by a third of people caring for a person with disability [19]. Our results indicate that this number could be higher in Mauritius. However, further investigation is required to confirm this finding.

Other studies support that caregiver burden increased during the COVID-19 lockdown [10-12,20]. However, they report the primary stressor as the absence of healthcare services. Lack of healthcare was not a significant factor for many of our participants, who instead cited geographical barriers, increased cost, and shortages of medication, and feeling trapped as primary stressors.

Our results indicate that participants received discouraging comments from healthcare workers, experienced low treatment efficacy, and desisted from treatment. The attitude of healthcare professionals has been shown to directly impact patient motivation and compliance [21,22]. Furthermore, access to successful role models and peer support increases health-seeking behaviours in neurological patients [23-25]. Our results show that due to the extent to which patients are isolated in Mauritius, they seldom interact with fellow patients and cannot benefit from peer support.

Unexpectedly, a significant portion of patients showed improvements in mood during lockdown due to increased interactions with family members. For these patients, pre-pandemic isolation acted as a protective factor as they experienced little disruption to their routines. These findings reflect the severity of patient isolation and are particularly pertinent as being employed and having social contacts is established as a protective factor for patient mental health [26,27]. Furthermore, these results strikingly contrast findings that in France, living with disability was a risk factor for poor mental health during the pandemic [28].

Our finding that increased contact with family members resulted in subjective gains in recovery is supported by a review study which established that a positive relationship exists between the quality of social contact and patient involvement in life situations [27]. The literature indicates that increased levels of emotional support [22], instrumental support [26], and informational support [27] improve functional status in activities of daily living, as was experienced by our patient group.

**Study limitations and strengths:** the qualitative design enabled a nuanced exploration of the lived experiences and challenges faced by individuals with neurological disabilities and their caregivers in a low-resource context. However, the study had several limitations. Language diversity, at times, constrained the depth of interviews. Cultural stigma surrounding injury and suspicion of being recorded led some participants to decline participation. Additionally, the context-specific nature of the study may limit transferability to other regions.

## Conclusion

This study highlights the complex and multifaceted barriers faced by Mauritians with neurological injuries and their family caregivers throughout the process of reintegration and recovery. Stigma, shame, and religious beliefs often result in social exclusion, while structural challenges-including inaccessibility, financial strain, and inadequate post-acute care-impede rehabilitation. Negative interactions with health professionals further undermine treatment adherence and motivation. Notably, some patients reported improved well-being during COVID-19 lockdowns due to increased familial presence, underscoring the extent of their typical isolation. Conversely, caregivers experienced heightened burnout, lack of respite, and diminished support. The six themes identified-caregiver burnout, barriers to treatment, poor social support, geographical barriers, financial burden, and isolation-provide a valuable foundation for future research, patient-centered surveys, and policy responses. These findings illustrate the urgent need for culturally responsive, community-based interventions that address the intersecting needs of patients and caregivers in low-resource settings.

***What is known about this topic***

- *There is a high incidence of non-communicable diseases in Mauritius, with an 80% increase in the rate of stroke between 2009-2019;*
- *While the provision of neurorehabilitation services has accelerated in the last five years, the island faces limited financial and human resources.*

***What this study adds***

- *Mauritians living with neurological disability and their family members often face cultural barriers to reintegration within the community;*
- *They are highly isolated due to financial, cultural, treatment, and geographical barriers. As a result, a significant portion showed improved mood and function during the COVID-19 lockdowns due to increased contact time with family members who were now permanently at home;*
- *Family caregivers of Mauritians with neurological disability face burnout as they take on multiple roles, which impacts their freedom and identity.*
